# Impact of Different Fertilizer Sources under Supplemental Irrigation and Rainfed Conditions on Eco-Physiological Responses and Yield Characteristics of Dragon’s Head (*Lallemantia iberica*)

**DOI:** 10.3390/plants12081693

**Published:** 2023-04-18

**Authors:** Saeid Heydarzadeh, Carmen Arena, Ermenegilda Vitale, Amir Rahimi, Mohsen Mirzapour, Jamal Nasar, Oscar Kisaka, Sumit Sow, Shivani Ranjan, Harun Gitari

**Affiliations:** 1 Department of Plant Production and Genetics, Faculty of Agriculture and Natural Resources, Urmia University, Urmia P.O. Box 165-57153, Iran; 2 Department of Biology, University of Naples Federico II, 80126 Naples, Italy; 3 NBFC-National Biodiversity Future Center, 90133 Palermo, Italy; 4 Department of Agricultural Biotechnology, Faculty of Agriculture, Siirt University, Siirt P.O. Box 56100, Turkey; 5 Guangxi Key Laboratory of Agro-Environment and Agro-Products Safety, National Demonstration Center for Experimental Plant Science Education, Agricultural College of Guangxi University, Nanning 530004, China; 6 Department of Agroforestry and Rural Development, University of Kabianga, Kericho P.O. Box 2030-20200, Kenya; 7 Department of Agronomy, Dr. Rajendra Prasad Central Agricultural University, Pusa 848125, Bihar, India; 8 Department of Agricultural Science and Technology, School of Agriculture and Environmental Sciences, Kenyatta University, Nairobi P.O. Box 43844-00100, Kenya

**Keywords:** antioxidant, drought, organic fertilizer, supplemental irrigation, sustainable agriculture

## Abstract

The effects of the irrigation regime and different fertilizer sources on the eco-physiological responses and yield characteristics of dragon’s head were explored in a factorial experiment based on a randomized complete block design with 12 treatments and 3 replications in the 2019 growing season. The treatments included six different fertilizer sources (animal manure, vermicompost, poultry manure, biofertilizer, chemical fertilizer, and control) and two irrigation regimes (rainfed and supplemental irrigation). The results indicated the positive effects of supplementary irrigation and the application of vermicompost, poultry manure, and animal manure by increasing the absorption of nutrients (phosphorus and potassium) and improving relative water contents, chlorophyll and carotenoid contents, and the fixed oil percentage of dragon’s head. The activities of catalase, ascorbate peroxidase, and superoxide dismutase decreased in the rainfed plants, whereas organic fertilizer application increased the antioxidant enzyme activity. The highest grain yield (721 kg ha^−1^), biological yield (5858 kg ha^−1^), total flavonoids (1.47 mg g^−1^ DW), total phenol (27.90 mg g^−1^ DW), fixed oil yield (200.17 kg ha^−1^), and essential oil yield (1.18 kg ha^−1^) were noted in plants that were treated with vermicompost under supplemental irrigation. Therefore, it is recommended that organic fertilizers such as vermicompost and poultry manure be used to substitute chemical fertilizers. These practices can help popularize organic crops using rainfed and supplementary irrigation.

## 1. Introduction

The increasing attention to the adverse effects of chemical pharmaceuticals, in comparison with the fewer effects associated with the use of organic herbal medications, has sparked an interest in the cultivation of medicinal plants [1]. Among the most common medicinal plants, dragon’s head (*Lallemantia iberica*, Lamiaceae family) is an important flowering and medicinal plant in Iran [2] that has antioxidant, antibacterial, and analgesic effects [3]. Dragon’s head is mainly grown as a rainfed crop and can tolerate mild drought conditions. However, its yield is drastically reduced when drought conditions worsen.

In arid and semi-arid environments, water is the most limiting factor in reducing agricultural crop production [4]. In rainfed farming, plants’ responses to water-deficit stress are affected by the varying frequency of dry and wet periods, the patterns of soil and atmospheric water deficits, and the degree and timing of droughts [5]. Dragon’s head is mainly cultivated under rainfed conditions and is moderately drought-tolerant, but its yield sharply decreases with rising drought conditions [6]. Rahimzadeh and Pirzad [7] indicated that water-deficit stress decreased chlorophyll content, catalase, ascorbate peroxidase, and superoxide dismutase activities, grain yield, biological yield, and seed oil content of flax in comparison to the control. Indeed, supplemental irrigation warrants the optimal use of rainfall where the minimal water stores of a locale can supply the dampness prerequisites of the plants at appropriate periods [8]. Accordingly, supplemental irrigation, which consumes little water, is helpful during the essential plant development and growth stages. It increases yield relative to the amount of water used [9]. It can decrease the effect of water stress on crop growth, development, and production and improve crop water productivity, mainly where irrigation supplements natural precipitation [10]. Mehrabi et al. [11] stated that the application of supplemental irrigation and biofertilizers on cumin plants can ameliorate the negative effects of water scarcity by boosting leaf water potential, the efficiency of carbon dioxide use, the transpiration rate, nutrient availability, and the water supply to the roots. Moreover, cumin plants’ growth, development, and dry-weight yield can also improve under water-deficit stress.

Although the use of biological and organic fertilizers in agriculture has been in place for decades to achieve clean production and sustainable cultivation [12,13], the latter has recently attracted consideration because of the acknowledgement of the damaging impacts of industrial fertilizers and the thoughtful consideration of organic and sustainable farming [14]. Without a serious focus on soil biodiversity, achieving organic and sustainable agriculture’s aims will not be easy [3]. Thus, in agricultural systems, the application of microorganisms is of strategic interest to reduce chemical fertilizer usage and improve environmental sustainability [12,15]. A biofertilizer is described as a substance that includes living microorganisms. It is recognized for promoting root growth and development besides playing a pivotal role in increasing germination rate and vigor in young plants, resulting in improved plant growth, development, and assimilation under water-shortage stress [8,15]. Animal manures supply the plant with macro- and micronutrient requirements while increasing organic matter in the soil, the relative C: N balance, and plant nutrient absorbability, all of which contribute to higher plant growth [16,17]. Another potential advantage of animal manure is its ability to reduce water-deficit stress. In such cases, plants alleviate the effects of water-deficit stress by adjusting osmotic pressure, which is achieved by regulating water uptake and cellular swelling [18]. Two fundamental properties of poultry manure are its ability to release nutrients slowly and its residual effect on crops. Fixed nutrients increase soil humus build-up and nutrient supplies in the soil, which improves uptake and the carrying of nutrients required inside the plant’s vessel system during water-shortage stress [19]. Vermicompost provides various benefits to agricultural soil, such as increasing its nutrient-holding capacity, retaining moisture, improving soil structure, increasing soil porosity, maintaining average soil temperature, improving nutrient content, increasing microbial activity, and enhancing antioxidant traits in plants and their tolerance to environmental stress [20]. Therefore, organic fertilizers improve soil chemical-compound attributes such as CEC, pH, and nutrient element accessibility to boost the level of organic matter in the field soil and hence the fertility of the earth [17]. Heydarzadeh et al. [5] stated that smooth vetch plants inoculated with biofertilizers improved non-enzymatic and enzymatic antioxidants, reducing water-stress damage. Maddahi et al. [12] showed that dragon’s head’s growth and nutritional conditions could affect its antioxidant properties. As a result, the winter-sown plants fertilized with vermicompost and biofertilizer had higher antioxidant properties. Darakeh et al. [21] noted that bio-organic fertilizers increased the phenol and flavonoid content of black cumin seeds. Ahmadian et al. [22] showed that the consumption of 20 tons of animal manure per hectare reduced the negative effects of drought stress by providing nutrients such as N, P, and K and improved the quality of cumin essential oil. Keshavarz et al. [23] indicated that mint plants treated with vermicompost and chemical fertilizers presented higher fixed oil percentage, fixed oil yield, essential oil, essential oil yield, and growth characteristics compared with the control. It has been reported that the use of organic fertilizers reduced the use of chemical fertilizers, supplied nutrients in a way entirely appropriate for the natural nutrition of plants, helped to preserve the environment, improved the fertility of agricultural lands, and increased the yield of cumin plants [11]. In addition, organic fertilizers improved crop growth and dry weight and increased the resistance of plants to water-shortage conditions, diseases, and pests [22].

Rainfall fluctuations and pollution from industrial fertilizers and pesticide residues are the two significant challenges in producing medicinal plants, whose production has significantly decreased in Iran. Hence, supplying such crops with organic and biological fertilizers is vital for improving their qualitative and quantitative yields under water stress [24]. Furthermore, introducing such organic and biofertilizers to crop growers and providing advice on substituting chemical fertilizers with environment-friendly ones can help organic crops expand under rainfed and supplementary irrigation conditions. Therefore, this research aimed to evaluate a novel aspect: the impact of organic, biological, and chemical fertilizers on the physiological responses, antioxidant activity, and yield of dragon’s head, a plant largely used in traditional medicine, under supplemental irrigation and rainfed conditions. The findings of this study can be used to improve the management approaches currently applied in the cultivation of dragon’s head to enhance its production.

## 2. Results

### 2.1. Photosynthetic Pigments, Relative Water Content (RWC), and Antioxidant Enzyme Activity

The photosynthetic pigments’ content (chlorophyll a (Chl a), chlorophyll b (Chl b), chlorophyll a+b (Chl a+b), and carotenoids (Car)) and relative water content (RWC) of dragon’s head were only influenced by the simple effects of the irrigation conditions and different fertilizer source treatments (Table 1). Specifically, photosynthetic pigments and RWC showed higher values under supplemental irrigation conditions (Table 1). Concerning the fertilization source, these traits were significantly promoted by vermicompost and poultry manure (Table 1). The activities of the CAT, SOD, and APX were influenced by the irrigation conditions, different fertilizer sources, and their interaction (Table 1). Supplemental irrigation increased enzyme activity regardless of the type of fertilizer (Table 1). Finally, among the different fertilizers, vermicompost determined the highest CAT, SOD, and APX activities (Table 1).

**Figure 1:**
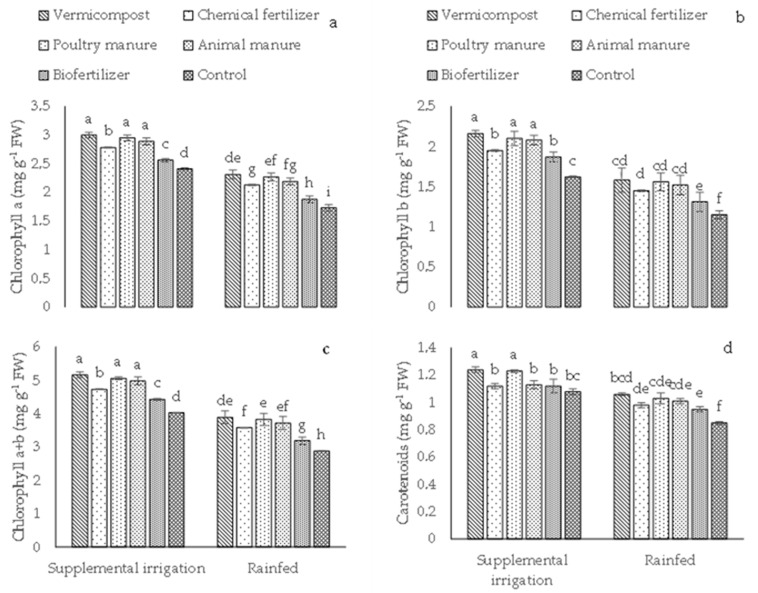
Means (±Standard deviation, SD) comparison for the interaction impacts of fertilizer and irrigation conditions on chlorophyll a (**a**), chlorophyll b (**b**), chlorophyll a+b (**c**), and carotenoids (**d**). FW: fresh weight. The means with unlike letters vary significantly at *p* ≤ 0.05 based on Tukey’s HSD test.

Different fertilizer sources significantly increased the photosynthetic pigment content compared to control plants in both rainfed and supplemental irrigation conditions (Figure 1a–d). The content of chlorophyll (a, b), chlorophyll a+b, and carotenoids was higher under supplemental irrigation than in rainfed conditions (Figure 1a–d). The highest concentrations of Chl a, Chl b, Chl a+b, and Car (3, 2.16, 5.16, and 1.24 mg g^−1^ FW, respectively) were recorded in plants treated with vermicompost under supplemental irrigation (Figure 1a–d). No difference in Chl (a, b) or Chl a+b content was observed among the poultry, animal manure, and vermicompost treatments under supplemental irrigation (Figure 1a–c). Furthermore, poultry manure and vermicompost application determined comparable Car content under supplemental irrigation (Figure 1d). Finally, the lowest concentration (in mg g^−1^ FW) of chlorophyll a (1.73), chlorophyll b (1.15), chlorophyll a+b (2.88), and carotenoids (0.85) was observed in control plants under rainfed conditions (Figure 1a–d).

**Figure 2:**
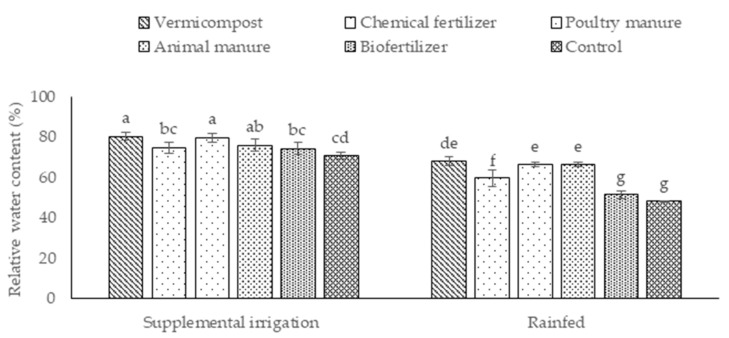
Means (±Standard deviation, SD) comparison for the interaction impacts of fertilizer and irrigation conditions on relative water content. The means with unlike letters vary significantly at *p* ≤ 0.05 based on Tukey’s HSD test.

Plants under supplemental irrigation exhibited a higher RWC than those under rainfed conditions (Figure 2). The highest RWC was recorded under supplemental irrigation in plants treated with vermicompost and poultry manure (Figure 2). Conversely, the lowest values were found under rainfed conditions in the biofertilized and control plants (Figure 2).

**Figure 3:**
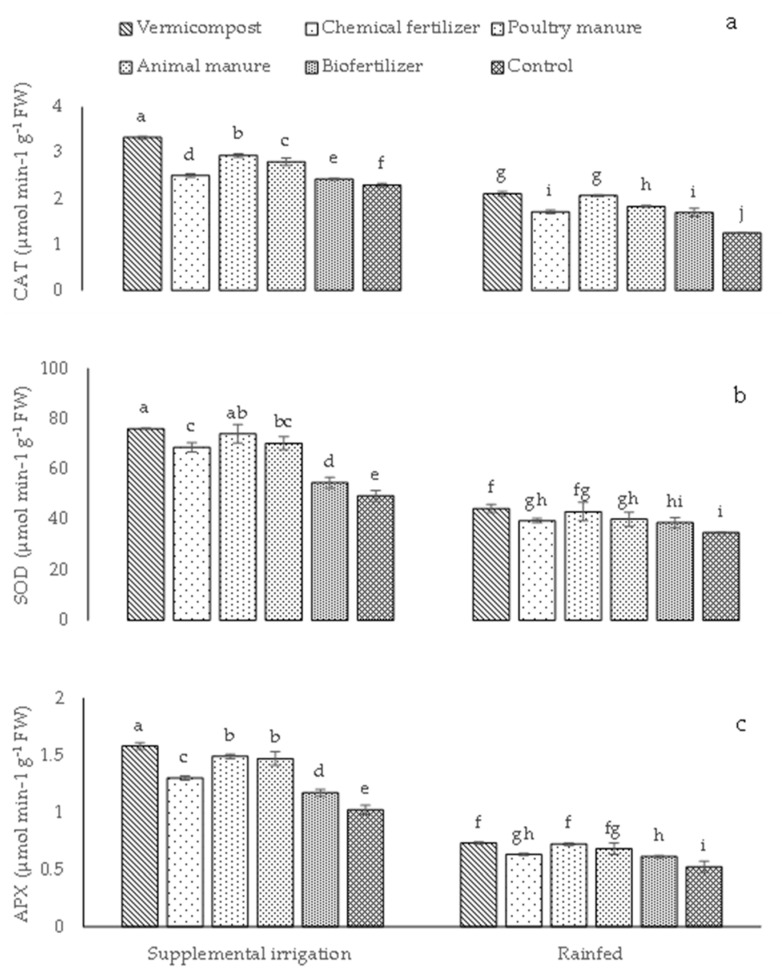
Means (±Standard deviation, SD) comparison for the interaction impacts of fertilizer and irrigation conditions on CAT, catalase activity (**a**); SOD, superoxide dismutase activity (**b**); and APX, ascorbate peroxidase activity (**c**). Means with same letters are not significantly different based on Tukey’s HSD test (*p* ≤ 0.05).

Applying different sources of fertilizers increased antioxidant enzyme activity under both rainfed and supplemental irrigation conditions (Figure 3a–c). CAT, APX, and SOD showed the highest activities (3.34, 76.04, and 1.58 μmol min^−1^ g^−1^ FW, respectively) in plants receiving supplemental irrigation and treated with vermicompost. Moreover, under supplemental irrigation conditions, no difference in SOD and APX activities was found between the poultry manure and animal manure treatments (Figure 3b–c). On the other hand, the lowest activities (1.25, 34.73, and 0.52 μmol min^−1^g^−1^ FW) were measured in control plants under rainfed conditions (Figure 3a–c). Lastly, no difference in CAT, SOD, and APX activities was found between vermicompost and poultry manure plants under rainfed conditions (Figure 3a–c).

### 2.2. Total Flavonoids and Total Phenol Content, DPPH Radical Scavenging, Superoxide Radical Scavenging, and Chain-Breaking Capacity

Irrigation conditions, fertilizer sources, and their interactions significantly influenced the total flavonoid and phenol content. TPC and TFC were promoted under supplemental irrigation (Table 2). Among fertilizers, the highest values were achieved in plants treated with vermicompost.

DPPH (-*2,2-diphenyl-1-picrylhydrazyl*) radical scavenging capacity, superoxide radical scavenging capacity, and chain-breaking capacity were only influenced by the simple effects of the irrigation conditions and different fertilizer treatments. Supplemental irrigation promoted DPPH RS, SRS, and CBAC more than rainfed conditions (Table 2). Among all fertilizer treatments, these specific traits were stimulated by the application of vermicompost and poultry manure (Table 2).

**Figure 4:**
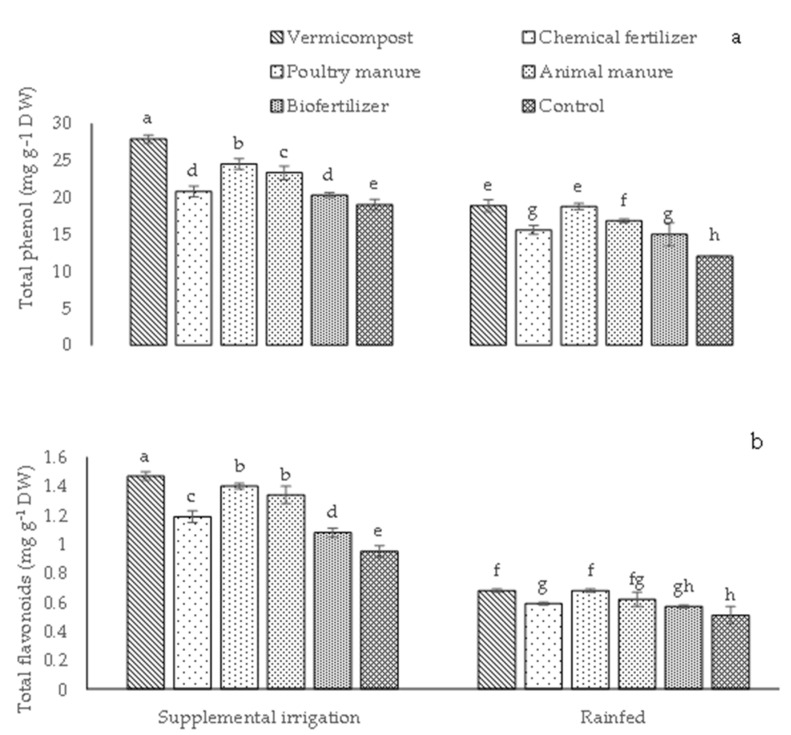
Means (±Standard deviation, SD) comparison for the interaction impacts of fertilizer and irrigation conditions on total phenol (**a**) and total flavonoids (**b**). DW: dry weight. The means with different letters differ significantly at *p* ≤ 0.05 based on Tukey’s HSD test.

The highest content of total flavonoids (1.47 mg g^−1^ DW) and total phenol (27.90 mg g^−1^ DW) was detected in plants treated with vermicompost under supplemental irrigation conditions. In contrast, the lowest values (12.06 and 0.51 mg g^−1^ DW) were recorded in control plants under rainfed conditions (Figure 4a, b). On the other hand, the application of poultry and animal manure induced comparable values of total flavonoids under supplemental irrigation conditions (Figure 4b). Similarly, in rainfed conditions, no difference was found in total flavonoids and phenols between vermicompost and poultry manure (Figure 4b).

**Figure 5:**
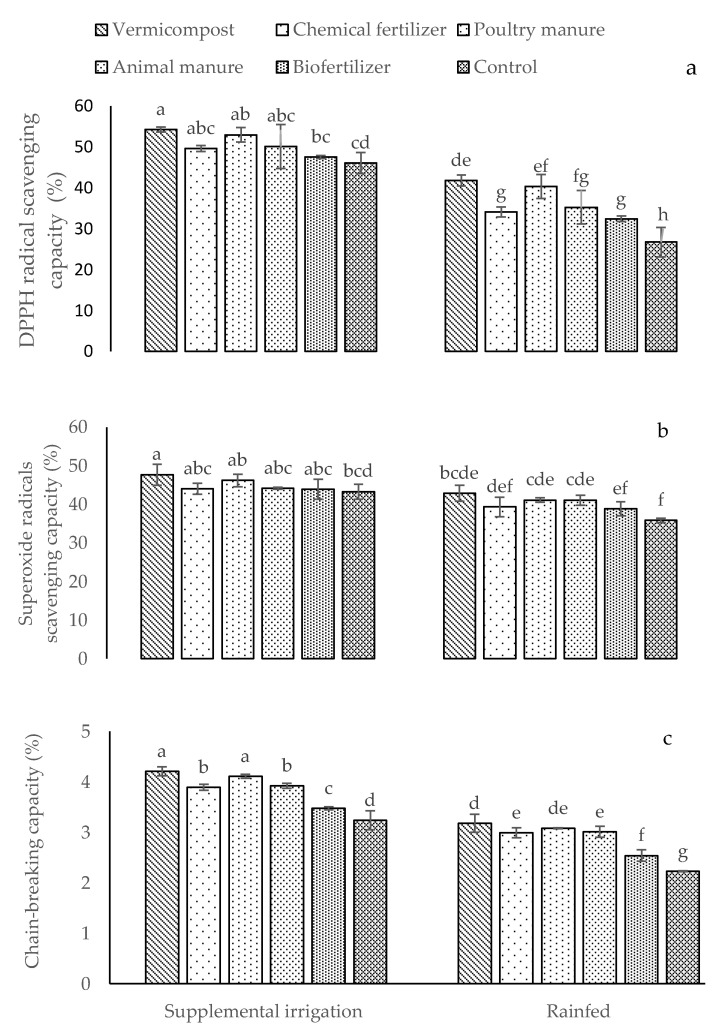
Means (±Standard deviation, SD) comparison for the interaction impacts of fertilizer and irrigation conditions on DPPH radical scavenging capacity (**a**), superoxide radical scavenging capacity (**b**), and chain-breaking capacity (**c**). DW: dry weight. The means with different letters differ significantly at *p* ≤ 0.05 based on Tukey’s HSD test.

The application of fertilizer sources significantly increased the percentages of DPPH radical scavenging capacity, superoxide radical scavenging capacity, and chain-breaking capacity versus the control (Figure 5a–c). The highest percentages of DPPH, superoxide radical scavenging capacity, and chain-breaking capacity (54.27, 47.74, and 4.21%, respectively) were observed in plants treated with vermicompost in supplemental irrigation conditions, while the lowest ones (26.74, 35.90, and 2.23%, respectively) were observed in control plants under rainfed conditions (Figure 5a–c). The application of poultry and animal manures did not affect the DPPH and superoxide radical scavenging capacities compared to the chemical fertilizers under supplemental irrigation (Figure 5a,b). Compared to chemical fertilizers, the animal manure treatment showed the same effect on chain-breaking capacity in supplemental irrigation conditions (Figure 5c). In addition, animal manure in rainfed conditions did not produce any effects on superoxide radical scavenging capacity compared to poultry manure (Figure 5b).

### 2.3. Content of Elements, Biological and Grain Yields, Fixed Oil Percentage and Fixed Oil Percentage Yield, and Essential Oil and Essential Oil Yield

The irrigation conditions, different fertilizer source treatments, and their interaction influenced the grain yield, the biological yield, and the essential oil yield (Table 3). On the other hand, the fixed oil percentage, fixed oil yield, essential oil, and the content of phosphorus and potassium were only influenced by the simple effects of the irrigation conditions and different fertilizer source treatments (Table 3). Supplemental irrigation promoted BY, GY, FO, FOY, EOY, and P and K content compared to rainfed conditions, while an opposite trend was observed for EO (Table 3). As regards fertilizer sources, plants receiving vermicompost exhibited the highest values of BY, GY, FO, FOY, EOY, EO, and P and K content, while the lowest values were obtained in control plants (Table 3).

**Figure 6:**
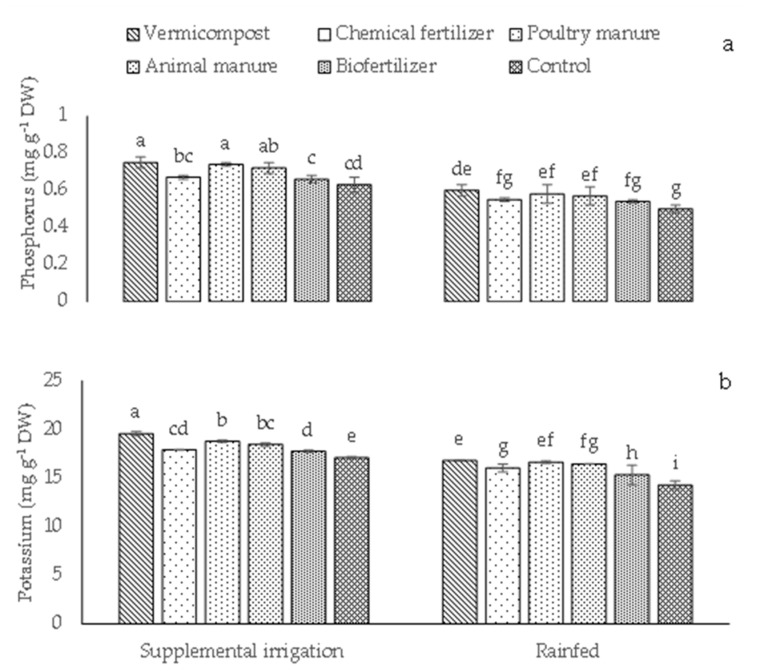
Means (±Standard deviation, SD) comparison for the interaction impacts of fertilizer and irrigation conditions on phosphorus (**a**) and potassium (**b**). DW: dry weight. The means with different letters differ significantly at *p* ≤ 0.05 based on Tukey’s HSD test.

The phosphorus and potassium contents in irrigated plants were higher than in rainfed plants (Figure 6a,b). The highest phosphorus and potassium contents were 0.75 and 19.56 mg g^−1^DW, respectively, recorded in irrigated plants treated with vermicompost, while their lowest values (0.50 and 14.28 mg g^−1^ DW, respectively) were found in rainfed control plants (Figure 6a,b). No significant difference in phosphorus in supplemental irrigation conditions between vermicompost and poultry manure was found (Figure 6a).

**Figure 7:**
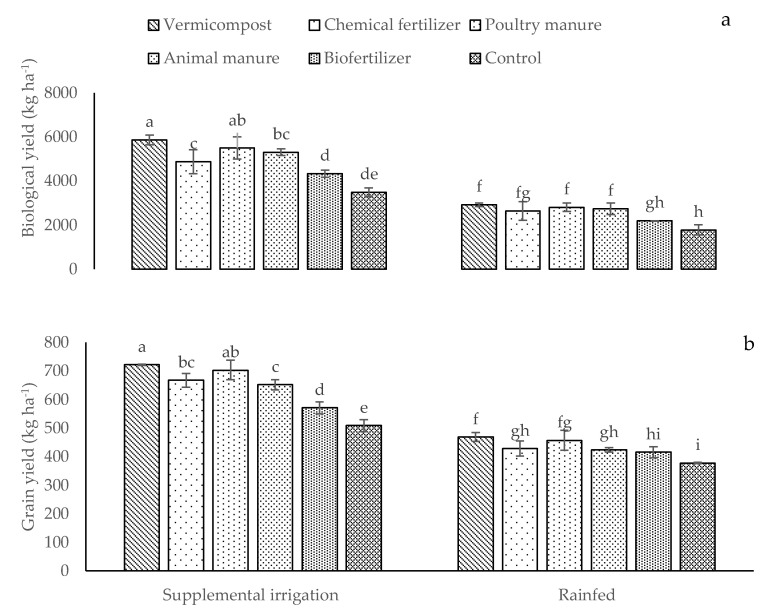
Means (±Standard deviation, SD) comparison for the interaction impacts of fertilizer and irrigation conditions on biological (**a**) and grain (**b**) yields. The means with dissimilar letters vary significantly at *p* ≤ 0.05 based on Tukey’s HSD test.

Biological and grain yields in supplemental irrigated plants were significantly greater than those under rainfed conditions (Figure 7a,b). Moreover, the different fertilizer sources applied under irrigation conditions considerably increased BY and GY compared to the control plots (Figure 7a,b). The highest BY and GY (5858 and 721 kg ha^−1^, respectively) were measured in plants treated with vermicompost and supplemental irrigation (Figure 7a,b). Conversely, their respective lowest values (1781 and 377 kg ha^−1^) were found in rainfed control plants (Figure 7a,b).

**Figure 8:**
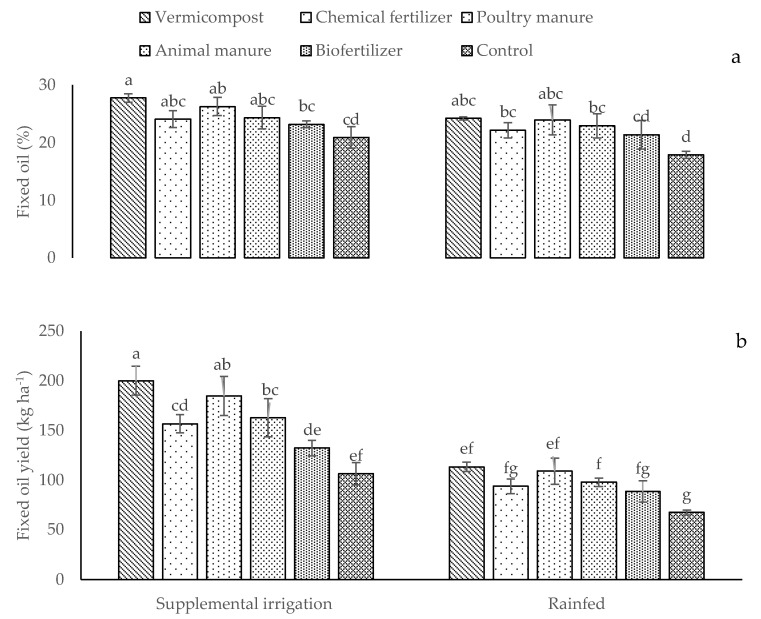
Means (±Standard deviation, SD) assessment for the interaction impacts of fertilizer and irrigation conditions on fixed oil percentage (**a**) and fixed oil yield (**b**). The means with unlike letters vary significantly at *p* ≤ 0.05 based on Tukey’s HSD test.

Fixed oil percentage and fixed oil yield of dragon’s head seeds improved considerably in plants treated with supplemental irrigation (Figure 8a,b). The highest FO (27.74%) and FOY (200.17 kg ha^−1^) were measured in irrigated plants treated with vermicompost. Conversely, the lowest FO and FOY (17.90% and 67.49 kg ha^−1^, respectively) were recorded in control plants under rainfed conditions (Figure 8a,b). Under supplemental irrigation conditions, poultry and animal manure and chemical fertilizer induced comparable FOs (Figure 8a), while under rainfed conditions, plants treated with vermicompost and poultry manure showed comparable FOs (Figure 8a). Finally, only organic fertilizers improved FOY compared to control plants under rainfed conditions (Figure 8b).

**Figure 9:**
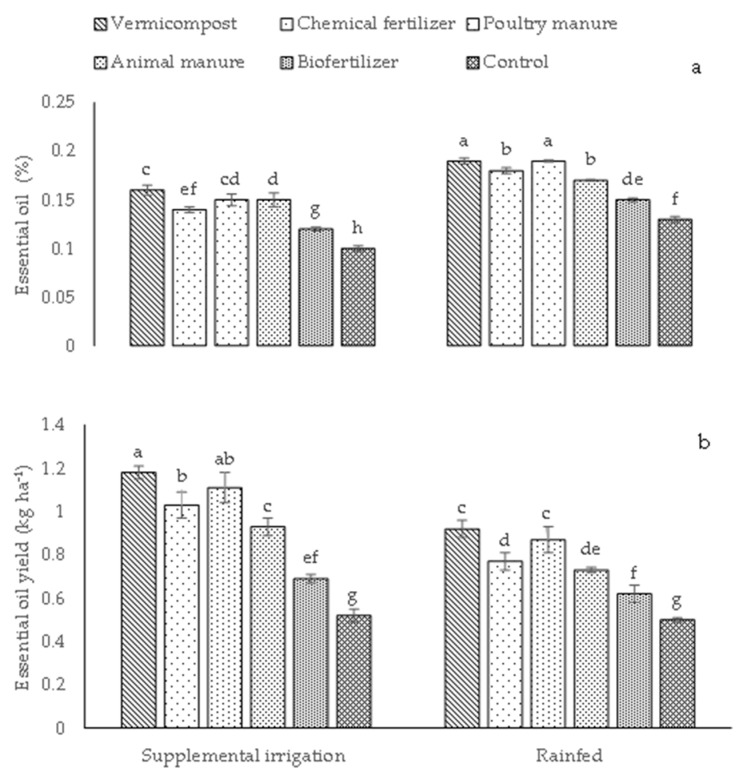
Means (±Standard deviation, SD) comparison for the interaction impacts of fertilizer and irrigation conditions on essential oil (**a**) and essential oil yield (**b**). The means with different letters differ significantly at *p* ≤ 0.05 based on Tukey’s HSD test.

The essential oil content in plants under rainfed conditions was higher than under supplemental irrigation conditions (Figure 9a). The highest EO (0.19%) was found in plants treated with vermicompost and poultry manure under rainfed conditions (Figure 9a). The lowest content (0.10%) was found in control plants under supplemental irrigation conditions (Figure 9a).

EOY (1.18 kg ha^−1^) achieved its highest value in plants treated with vermicompost and poultry manure under supplemental irrigation conditions (Figure 7b). In contrast, the lowest EOY (0.50 kg ha^−1^) was recorded in rainfed control plants (Figure 7b). Finally, EOY did not show significant differences between plants treated with vermicompost and poultry manure under rainfed conditions (Figure 7b).

## 3. Discussion

Based on our findings, by enhancing water-shortage stress in dragon’s head plants, chlorophyll a and b, chlorophyll a+b, and carotenoids at flowering stages were decreased. Nevertheless, they improved in response to different fertilizer sources. The degradation of such pigments or the reduction in their synthesis, associated with the reduced activity of enzymes involved in their synthesis, causes chlorophyll decline in crops exposed to water-deficit stress, resulting in reduced assimilation material and, thus, performance losses [25]. Because chlorophyll and carotenoids are always bound to proteins, fertilizer application delivers the nitrogen requirements of photosynthetic pigments and plant cell proteins, leading to an enhancement in the amount of these pigments in crops [25,26]. As a result of this increase, crop productivity rises.

Darakeh et al. [21] maintain that the leaf RWC of dragon’s head diminishes as water stress increases. The decrease in tissues’ turgor in plant and leaf RWC can be the first effect of water-deficit stress, which can have a natural influence on the development, growth, and ultimate size of cells [27]. Organic fertilizer enhances water uptake in the host plant by altering root architecture and spreading the plant’s root system [28]. Indeed, organic fertilizers can alleviate the negative influences of water-deficit stress on plants by enhancing the moisture potential of the leaf, the transpiration rate, the photosynthetic efficiency, and the rate of CO_2_ use. Moreover, they can promote the absorption of nutrients, enhancing growth and plant production [28].

Under rainfed and supplemental irrigation conditions, different sources of fertilizers enhanced antioxidant enzyme activity. In agreement with current findings, Ebrahimi et al. [29] reported improved antioxidant enzyme activity in eggplants under deficit irrigation when treated with organic fertilizers. The low CAT activity, particularly under moisture-shortage pressure conditions, may also be attributed to its inactivation. Moreover, as noted by Hameed et al. [30], such a decrease in the activity of CAT could result from the prevention of photorespiration and photosynthesis under moisture-shortage pressure. Figure 3 shows that APX activity decreased remarkably in plants grown under rainfed conditions. Drought tolerance is associated mainly with its suitable manipulation of antioxidant production, supporting the current results [28]. Previous studies have indicated that during water-deficit stress, excessive concentrations of H_2_O_2_ may inhibit or down-regulate such antioxidant enzymes [5,7]. Therefore, this suggests that bio-organic fertilizers under rainfed conditions could lessen plant ROS damage. In particular, in inoculated plants, SOD activity converts O_2_^−^ to H_2_O_2_ [7]. Under conventional (rainfed) conditions, SOD activity improves considerably in plants treated with bio-organic fertilizers such as vermicompost, poultry, and animal manure.

The application of different sources of fertilizers raised the content of total flavonoids and total phenols in dragon’s head plants in both irrigation systems. In addition, it is stated that vermicompost fertilizers improved antioxidant compound synthesis in peppermint plants in irrigation states [31]. Plant flavonoids and phenols are thought to play a significant function in plants as security combinations against different reactive oxygen species (ROS) combinations and the damage of free radicals [12]. Generally, biofertilizers could change the metabolite compounds due to gibberellins or cytokines [21]. This enhancement can depend on more available nutrients and their uptake by plants following the decrease in pH after applying biofertilizers [32]. Furthermore, it was discovered that fertilizer treatments were efficient in DPPH radical suppression, as it was observed with the highest DPPH antioxidant activity obtained using vermicompost [21,33]. As noted by Kumar et al. [34], free radicals, such as superoxide, can oxidize numerous metabolic pathways and harm the physiological function of the plant. Such free radicals can be disposed of using antioxidant compounds that function as free radical scavengers [33]. It is worth mentioning that most of the antioxidant combinations that can counteract and scavenge the ROS appear (in plant extracts) in the polar stage [34]. Breaking chain reactions of such free radicals is a primary strategy under low concentrations of phenolic combinations, metabolites, or other antioxidant combinations. Such phenol combinations appear to be active plant substances that execute this function [35]. The antioxidant characteristics of dragon’s head were studied using organic fertilizers such as vermicompost and poultry and animal manure, and it was shown that organic fertilizers enhanced DPPH radical scavenging percentage, total phenol content, and chain-breaking activity in comparison with the control in both irrigation regimes. Such upsurges may be attributed to the functional role of bio-organic fertilizers in enhancing antioxidant combinations that inhibit ROS associated with oxidative tension. The application of bio-organic fertilizers under irrigation conditions subsequently contributed to an increased rate of net photosynthesis in plants and an increased action of the enzymes implicated in the biosynthesis of starch and protein in the synthesis of secondary metabolite combinations [17,31]. Because hydrocarbons are the structure required to biosynthesize phenol combinations, an enhancement in their concentration leads to an increase in the material for phenol combinations. This increment may be related to the assignment of additional carbons to the shikimate route and may contribute significantly to the synthesis of flavonoids and phenols [31].

As the water deficit increases, nutrient availability usually decreases [36]. Therefore, given the lower nutrient content of the seeds under rainfed conditions, it can be concluded that nutrient uptake potential was low in rainfed plants due to a rise in water deficit. Such results align with the observation made by Kulak et al. [37] that nutrient solubility decreases with soil moisture. Furthermore, diminished moisture absorption reduces transpiration and photosynthesis from a physiological standpoint [8]. Additionally, active mobilization procedures are disrupted under such conditions to conserve biological energy consumption. All these mechanisms result in a substantial decrease in root assimilation capacity, resulting in a subsequent reduction in nutrient absorption [38]. It has been previously noted that organic and biological fertilizers enhance plant development and growth, nutrient absorption, and photosynthetic efficiency considerably due to the increased activity of alkaline phosphatase and acid phosphatase [39]. The application of bio-organic fertilizers impacts physiological processes by promoting enzymes and the transfer of photosynthetic products, as well as by stimulating the division and elongation of cells, which lead to increased growth and mineral content in leaves [8,17].

In this study, applying different fertilizer sources improved the biological and grain yields of dragon’s head plants compared to those not subjected to fertilization under both irrigation systems. Supplemental irrigation at either the pod-filling or flowering phases of cumin improved biological products by positively affecting the plant’s height and the growth of extra branches [11]. Mirzamohammadi et al. [31] reported that vermicompost application improved the development and production of peppermint plants under water-shortage stress states by increasing nutrient accessibility and absorption. Rahimi et al. [17] reported that applying bio-organic fertilizers improved the efficacy of photosynthesis and enhanced the growth of *Syrian cephalaria* in rainfed conditions. Hosseinzadeh et al. [18] stated an enhancement in the biological and grain yield of purslane following the application of animal manure and biofertilizer. The authors attributed such results to the positive effect exerted by the treatments on vegetative growth, chlorophyll synthesis, and photosynthetic capacity, especially under water-shortage tension [29]. The beneficial influence of manure and vermicompost may be related to increased soil organic matter content and a regulated availability of nutrients in the agricultural soil, which directly influence plant photosynthetic and vegetative growth [17].

Given that fixed oil yield is the product of fixed oil percentage and grain yield, any aspect that affects these two traits can affect fixed oil yield. Similar findings indicating a reduced fixed oil percentage under water-deficit tension were observed by Heydari and Pirzad [6]. As a result, the use of bio-organic fertilizers not only increases the water potential of the leaf, the rate at which the plant utilizes transpiration, the carbon dioxide levels, and the production of growth stimulants but also leads to root development besides enhancing water uptake in the plant during water-shortage tension and increasing the percentage of dragon’s head fixed oil [6,21]. Rahimi et al. [17] noted that using vermicompost and animal manure improved the uptake of water and nutrients in *Syrian cephalaria* plants, leading to an increase in seed fixed oil percentage and fixed oil yield. It has been reported that organic and biological fertilizers positively impact photosynthetic products, mainly carbohydrates. The export of carbohydrates from photosynthesizing leaves to seeds may also have increased the fixed oil percentage given that carbohydrates are precursors of fatty acid biosynthesis pathways [21,37].

Hosseinzadeh et al. [18] reported that applying organic and biological fertilizers improved the essential oil content and yield of purslane plants with low irrigation. Additionally, such treatments may enhance the extent of essential oil-producing glands in the flowers and leaves of the dragon’s head plant under rainfed conditions. Essential oils are terpenoids, requiring ATP, Acetyl-CoA, and NADPH for synthesis [40]. Therefore, essential oil biosynthesis is entirely dependent on the supply of plant mineral nutrients [41]. In the current investigation, organic fertilizers such as vermicompost and poultry manure may have enhanced nutrient uptake and improved plant moisture relationships compared to control plants, leading to essential oil increment. It has been stated that bio-organic and chemical fertilizers and vermicompost enhanced the amount and quality of essential oils produced in coriander and Moldavian balm [11,41]. Under water-scarcity stress, the yield of essential oils increases and/or decreases induced by the relations between the raised essential oil percentage and the diminished yield [40]. The employment of organic fertilizers such as vermicompost represented an environmentally friendly tactic to improve the sustainable production of bioactive molecules in dragon’s head plants.

## 4. Materials and Methods

### 4.1. Experimental Design

The experiment was carried out at the research farm of the Medicinal Plants and Drugs Research Institute of the Urmia University, located in West Azerbaijan, Iran (44°58′12.42″ E, 37°39′24.82″ N, and 1338 m elevation). The experiment was performed in a factorial form based on a randomized complete block design with six different fertilizer sources (animal manure, vermicompost, poultry manure, biofertilizer, chemical fertilizer, and control) and an irrigation regime at two levels (rainfed and supplemental irrigation) in three replicates in the 2019 growing season. The plants were planted with a row-to-row distance of 40 cm and an interseed spacing of 3 cm. The 99% purity and 98% vigor seeds were planted in mid-March. At the beginning of the experiment, the soil at the upper depth of 0–0.3 m had a loam-clay texture with a N content of 0.54%, a pH of 7.9, K contents of 407, and P contents of 10.1 mg kg^−1^. The climatic conditions of the experimental site were characterized by an average temperature for the planting period (March–July) of 15.7 °C and a total rainfall of 146.7 mm. Most of the rainfall was well distributed for the early vegetative growth of the dragon’s head plants.

### 4.2. Plant Material

Before sowing, dragon’s head seeds were inoculated with 108 CFU (-Colony-Forming Unit per mL) mL^−1^ bacterial population of *Azotobacter chroococcum* nitrogen-fixing bacteria and fertile phosphate-2 (containing two types of phosphate-soluble bacteria of the species *Bacillus lentus* and *Pseudomonas putida*) at a rate of 2 L ha^−1^ in the shade [42]. Then, the seeds were dried in the shade for half an hour before sowing. Chemical fertilizer treatments were applied based on plant needs and soil decomposition results. First, 120 kg ha^−1^ of triple superphosphate and 60 kg ha^−1^ of urea fertilizers were added at the planting stage. At the first stage of stem formation, the plants were supplied with 60 kg ha^−1^ of urea fertilizers as a top dressing. The vermicompost (at 15 t ha^−1^), animal manure (at 20 t ha^−1^), and poultry manure (at 10 t ha^−1^) were added to the selected plots during land preparation practices and thoroughly mixed with soil. The characteristics of the organic fertilizers used in the study are displayed in Table 4.

At flower initiation, supplemental irrigation was supplied using the method of Benami and Ofen [43]. The amount of irrigation water needed to reach field capacity (FC) was 600 m^3^ ha^−1^. Leaves were sampled at the end of the flowering stage to determine physiological parameters. Fresh leaf samples were covered with aluminum foil, stored in nitrogen tanks, and stored in a freezer at −80 °C. All cultivation methods were carried out uniformly for all experimental treatments. All experimental treatments were harvested individually after reaching full maturity (in mid-July), paying attention to grain yield and biological yield from 10 plants per plot. The samples were dried in an oven for 24 h at 70 °C before being analyzed.

### 4.3. Measurements

#### 4.3.1. Photosynthetic Pigment Content

To determine the photosynthetic pigment content, namely chlorophyll a and b and carotenoids, 0.5 g of fresh leaves was ground in liquid nitrogen, blended with 10 mL of 80% acetone, and separated into supernatant and solid parts of the leaf sample (precipitate or pellet) by centrifugation at 4000 rpm for 15 min. Chlorophyll a and b and carotenoid content was measured using a spectrophotometer at the full flowering stage [44].

#### 4.3.2. Relative Water Content

Relative water content of leaf samples was measured following the method proposed by Khosravi et al. [45] (Equation (1)).

Relative water content = ((Fresh weight − dry weight)/(Turgid weight − dry weight)) × 100(1)

#### 4.3.3. Antioxidant Enzyme Extraction and Assays

For quantification of antioxidant enzyme activity, fresh material (100 mg) was ground in 2 mL of 0.1 M KH_2_PO_4_ containing 5% polyvinylpyrrolidone (PVP) and buffered at a pH of 6. Thereafter, the extracts were centrifuged at 3 °C for 30 min at 15,000 rpm, and the activity of the enzymes was estimated from the clear supernatant [46].

##### Catalase (CAT)

Catalase (CAT) activity was determined at 240 nm based on the variation in concentration of hydrogen peroxide (H_2_O_2_). In this case, the reaction mixture contained 1.9 mL of 50 mM K_3_PO_4_, which was buffered at a pH of 7.0. Enzymatic activity was then read over 60 s per mg of protein based on absorption variations [47].

##### Superoxide Dismutase (SOD)

Superoxide dismutase (SOD) activity was assessed at 560 to minimize the loss of nitroblue tetrazolium (NBT) photochemical as noted by Beyer and Fridovich [48]. In this study, one unit of SOD was taken as the quantity of enzyme that inhibits a 50% decrease in NBT.

##### Ascorbate Peroxidase (APX)

By employing the Nakano and Asada method [49], ascorbate peroxidase (APX) activity was measured with a reaction mixture containing 1 mL of 0.5 mM ascorbic acid, 1 mL 100 mM K_3_PO_4_ buffered at a pH of 7, 100 μL enzyme extract, and 0.1 mL H_2_O_2_ 0.1 mM. The absorption was then read with an absorbance coefficient of 2.8 mM^−1^ cm^−1^ at 290 nm.

#### 4.3.4. Antioxidant Compounds

Fresh leaves of dragon’s head were cut into small pieces and dried and powdered at room temperature in the shade. The methanolic extraction was performed with the addition of 25 mL solvent to 2 g sample and was shaken for 60 min at 1000 rpm. Then, the extract was passed through Whatman filter paper No. 1 (Whatman Ltd., Maidstone, UK). The solutions were then stored at 4 °C until the experiments. Light exposure was avoided during the extraction process [50].

##### Total Phenolic Content (TPC)

The total phenolic content was evaluated using the Folin–Ciocalteu technique [51,52]. A total of 1600 μL of distilled water and 10 μL of methanolic extracts were mixed and treated with 200 μL of Folin–Ciocalteau reagent (10% *v/v*), which was prepared in distilled water for 5 min at 25 °C. Thereafter, 200 μL of NaCO_3_ (7.5%) was added, and the mixture was kept at 25 °C in the dark for 30 min. For quantitative examination of TPC, the sample’s absorbance was measured using a UV/Visible spectrophotometer (DB-20/DB-20S) at 760 nm. TPC was calculated as mg of gallic acid (3,4,5-trihydroxybenzoic acid) g^−1^ dry weight using gallic acid as an external standard.

##### Total Flavonoid Content (TFC)

The aluminum chloride-based colorimetric method was used to evaluate the total flavonoid content in the methanolic extracts. In brief, 150 μL of sodium nitrate (5% *w/v*) was mixed with 30 μL of the methanolic extract, and after waiting for 5 min, 3 mL of aluminum chloride hexahydrate (10% *w/v*) was added and incubation was performed for 5 min. Thereafter, 1 mL of NaOH (1.0 M) was added, followed by dilution of the mixture with distilled water to the mark. After incubation in the dark for 30 min at 25 °C, the solution’s absorbance was measured in a spectrophotometer at 510 nm. The external standard for TFC quantification was Quercetin (QE), and TFC was reported as mg QE g^−1^ dry weight [51,52].

##### DPPH (2,2-diphenyl-1-picrylhydrazyl-hydrate) Radical Scavenging Activity

The DPPH radical scavenging activity of samples was measured using the colorimetric process as outlined by Brand-Williams et al. [53]. A total of 2.0 mL of DPPH solution was mixed with 15 μL of methanolic extract, and the mixture was incubated in the dark for 30 min at 20 °C. The solution’s absorbance was evaluated at 517 nm. The DPPH inhibition was computed using Equation (2).

Inhibition (%) = ((Ab_control_ − Ab_sample_)/(Ab_control_)) × 100 (2)

where Ab_control_ and Ab_sample_ are the absorbances of the control and the sample, respectively.

##### Superoxide Radical Scavenging Activity

For measurement of superoxide radical scavenging activity [54], 1 mL of extract was put into 9 mL of 5 mM Tris-HCl buffer (pH 8.2). Thereafter, to the same mixture, 40 μL of 4.5 mM pyrogallol was added. After shaking for 3 min, the solution’s absorption spectrum at 420 nm was evaluated using a spectrophotometer. Superoxide radical scavenging activity was computed, as indicated in Equation (3), as the expression of the oxidation degree of a test group in association with that of the control.

Superoxide radical scavenging (%) = ((Ab_0_ − Ab_1_)/(Ab_0_)) × 100 (3)

where Ab_1_ is the absorbance of the extract and Ab_0_ is the absorbance of the Tris-HCl buffer with pyrogallol.

##### Chain-Breaking Capacity

Chain-breaking capacity was measured using DPPH reagent and Brand-Williams et al.’s [53] method. A total of 10 μL of the extract was combined with 1.9 mL of DPPH methanolic solution (0.004%). Their uptakes at 0 h and after 30 min of incubation at room temperature and in darkness were evaluated at 515 nm in a spectrophotometer. The reaction rate was calculated according to the formula in Equation (4).

(4) $${A_{bs}}^{-3}-{A_{bs0}}^{-3}=-3kt$$

where A_bs0_ is the initial absorbance, A_bs_ is the absorbance at the increasing time (t), and the reaction rate is expressed as k. Antioxidant activity was reported as -A_bs_**^−^**^3^ /min/mg extract.

#### 4.3.5. Nutrients of Potassium and Phosphorus

To determine the nutrient content of leaf samples, dried leaf samples were milled, digested, and analyzed through combustion (4 h at 500 °C). The leaves’ ashes (5 mg) were digested in 1 mL of 2 N HCl, and by use of Whatman filter paper of grade 42, the extracts obtained were filtered. The samples were then filtered, and using the vanado-molybdate method, the phosphorus (P) content was determined calorimetrically. The method was based on observing the yellow color of the unreduced vanado-molybdo-phosphoric heteropoly acid suspended in HNO_3_ medium. The color intensity was determined at 470 nm using a Spectronic 20 colorimeter [55,56]. The amount of potassium (K) was measured by a flame photometer [55,56].

#### 4.3.6. Seed and Biological Yield Characteristics

All experimental treatments were harvested individually after reaching full growth maturity, paying attention to grain yield and biological yield from 10 plants plot^−1^. The plant samples were oven-dried for two days at 72 °C. Then, they were reweighed on a scale to find the difference.

#### 4.3.7. Content of Fixed Oil and Essential Oil

The essential oil content of dragon’s head grain was quantified using the Clevenger apparatus [57], whereas the fixed oil content of dragon’s head grain was extracted following the Soxhlet technique [58] using a methanol/chloroform organic solvent. Essential oil yield and fixed oil yield were computed using Equations (5) and (6).

Essential oil yield = Grain yield × Essential oil(5)

Fixed oil yield = Grain yield × Fixed oil percentage(6)

#### 4.3.8. Data Analyses

The data generated in this study were analyzed using the SAS 9.1 software. The effects of two independent factors, i.e., different fertilizer applications (F) and irrigation conditions (I), as well as their possible interaction on physiological processes, antioxidant enzyme activity, antioxidant compounds, element content, and plant yield, were assessed by two-way ANOVA. Means were compared by Tukey’s HSD at the *p* < 0.05 level. The graphs were drawn in Excel.

## 5. Conclusions

The evaluation of the physiological and biochemical characteristics of the dragon’s head plant subjected to supplemental irrigation conditions and different fertilizer sources showed that the application of vermicompost and poultry and animal manure was more effective compared to chemical fertilizers in improving the grain, fixed oil, and essential oil yields of the dragon’s head plant. The best plant performance under supplemental irrigation conditions was achieved through the improvement in nutrient absorption (phosphorus and potassium) and the relative water content, as well as an increase in photosynthetic pigment content, enzyme activity, and the percentage of inhibition of antioxidant radicals.

Such a result was the primary goal of our experiment, given the importance of the use of dragon’s head plants for nutraceutical and curative purposes. Our data demonstrated that even if the experimentation period was just one year, soil fertilization with vermicompost, and poultry and animal manure in rainfed conditions and supplemental irrigation are strongly recommended to increase the nutraceutical and physiological traits of the dragon’s head plant. This practice also effectively reduces the environmental pollution caused by the overuse of chemical fertilizers, achieving sustainable agricultural goals, and allows the cultivation of the dragon’s head plant with low water requirements, reducing water consumption.

Even if further investigations are needed to assess what different fertilizer sources may exert over the long term, our results are promising for the implementation of dragon’s head cultivation in arid and semi-arid regions.

## Figures and Tables

**Table 1:** Analysis of variance and comparison of means of physiological traits and antioxidant enzyme activity of dragon’s head plants in response to irrigation conditions (I), different fertilizer sources (F), and their interaction (I × F). Different letters in each column indicate significant differences according to Tukey’s HSD test (*p* < 0.05).

		Chl a	Chl b	Chl a+b	Car	RWC	CAT	SOD	APX
Irrigation conditions									
Supplemental irrigation		2.76 a	1.96 a	4.73 a	1.12 a	70.19 a	2.72 a	65.47 a	1.34 a
Rainfed		2.09 b	1.43 b	3.51 b	1.01 b	66.12 b	1.78 b	40.08b	0.65 b
Different fertilizer sources									
Vermicompost		2.65 a	1.87 a	4.52 a	1.19 a	77.48 a	2.72 a	59.57 a	1.15 a
Chemical fertilizer		2.46 c	1.80 a	4.24 b	1.03 bc	74.83 a	2.11 d	54.90 b	0.96 c
Poultry manure		2.61 ab	1.83 a	4.44 a	1.18 a	76.89 a	2.51 b	57.07 ab	1.11 b
Animal manure		2.54 b	1.70 b	4.26 b	1.08 bc	67.12 b	2.32 c	56.42 b	1.07 b
Biofertilizer		2.22 d	1.59 c	3.81 c	1.00 c	62.84 c	2.06 d	46.63 c	0.89 d
Control		2.07 e	1.38 d	3.46 d	0.93 d	49.46 d	1.77 e	42.03 d	0.77 e
Sources of variations	df	Mean Square							
Block (Repetition)	2	0.00004	0.02	0.01	0.002	13.59	0.0003	1.10	0.0001
Irrigation (I)	1	4.14 **	2.57 **	13.29 **	0.11 **	148.79 **	8.00 **	5804.66 **	4.26 **
Fertilizer (F)	5	0.32 **	0.19 **	1 **	0.06 **	690.54 **	0.69 **	282.99 **	0.12 **
I × F	5	0.0004 ^ns^	0.002 ^ns^	0.002 ^ns^	0.0008 ^ns^	6.95 ^ns^	0.04 **	110.07 **	0.02 **
Error	22	0.003	0.005	0.009	0.002	6.15	0.002	5.59	0.001
C.V (%)		2.44	4.42	2.32	4.62	3.63	2.14	4.48	3.44

^ns^ (not significant); ** (significant at *p* < 0.01); df (degrees of freedom); Chl a (chlorophyll a); Chl b (chlorophyll b); Chl a+b (chlorophyll a+b); Car (carotenoids); RWC (relative water content); CAT (catalase activity); APX (ascorbate peroxidase activity); SOD (superoxide dismutase activity).

**Table 2:** Analysis of variance and comparison of means of antioxidant activity traits of dragon’s head plants in response to irrigation conditions (I), different fertilizer sources (F), and their interaction (I × F). Different letters in each column indicate significant differences according to Tukey’s HSD test (*p* < 0.05).

		TPC	TFC	DPPH RS	SRS	CBAC
Irrigation conditions						
Supplemental irrigation		22.08 a	1.24 a	48.75 a	43.57 a	3.78 a
Rainfed		16.76 b	0.61 b	38.76 b	41.25 b	2.86 b
Different fertilizer sources						
Vermicompost		23.46 a	1.07 a	50.17 a	45.97 a	3.72 a
Chemical fertilizer		18.82c	0.89 c	45.20 bc	42.24 bc	3.46 b
Poultry manure		21.70 b	1.04 a	47.36 ab	45.08 a	3.64 a
Animal manure		21.06 b	0.98 b	44.92 bc	43.49 ab	3.44 b
Biofertilizer		17.66 d	0.83 d	42.47 c	40.27 c	3.01 c
Control		13.83 e	0.73 e	32.41 d	37.39 d	2.66 d
Sources of variations	df	Mean Square				
Block (Repetition)	2	1.10	0.0002	3.19	0.89	0.01
Irrigation (I)	1	255.30 **	2.54 **	898.40 **	48.51 **	7.54 **
Fertilizer (F)	5	70.75 **	0.24 **	225.44 **	60.91 **	1.00 **
I × F	5	5.48 **	0.12 **	2.07 ^ns^	1.08 ^ns^	0.002 ^ns^
Error	22	0.48	0.001	9.95	4.83	0.009
C.V (%)		3.57	4.37	4.58	5.18	2.88

ns (not significant); ** (significant at *p* < 0.01); df (degrees of freedom); TPC (total phenol content); TFC (total flavonoid content); DPPH RS (DPPH radical scavenging); SRS (superoxide radical scavenging); CBC (chain-breaking activity capacity).

**Table 3:** Analysis of variance and comparison of means of content of elements and yield traits of dragon’s head plants in response to irrigation conditions (I), different fertilizer sources (F), and their interaction (I × F). Different letters in each column indicate significant differences according to Tukey’s HSD test (*p* < 0.05).

		BY	GY	FO	FOY	EO	EOY	P	K
Irrigation conditions									
Supplemental irrigation		4629.15 a	636.65 a	24.40 a	157.07 a	0.14 b	0.91 a	0.66 a	18.26 a
Rainfed		2518.45 b	427.71 b	22.08 b	95.06 b	0.17 a	0.73 b	0.59 b	15.92 b
Different fertilizer sources									
Vermicompost		4394.90 a	594.62 a	25.97 a	155.28 a	0.18 a	1.05 a	0.74 a	18.19 a
Chemical fertilizer		3757.20 c	536.90 b	23.24 bc	131.98 b	0.15 d	0.83 d	0.62 c	16.96 a
Poultry manure		4153.60 ab	579.45 a	25.08 ab	143.73 ab	0.17 b	0.99 b	0.67 b	17.71 b
Animal manure		4026.70 bc	547.16 b	23.49 bc	128.13 bc	0.16 c	0.83 c	0.59 cd	17.45 b
Biofertilizer		2767.40 d	492.41 c	22.27 c	110.39 c	0.13 e	0.66 e	0.57 d	16.53 d
Control		2343 e	442.55 d	19.39 d	86.87 d	0.12 f	0.51 f	0.53 e	15.69 e
Source of variation	df	Mean Square							
Block (Repetition)	2	31900.32	93.36	0.89	44.35	0.00006	0.001	0.0006	0.28
Irrigation (I)	1	10,023,862.05 **	310,044.80 **	48.51 **	34607.78 **	0.008 **	0.27 **	0.04 **	49 **
Fertilizer (F)	5	1,024,266.40 **	29,355.85 **	31.97 **	3584.23 **	0.003 **	0.25 **	0.03 **	4.81 **
I × F	5	232,976.53 **	13,046.31 **	1.08 ^ns^	676.56 ^ns^	0.0001 ^ns^	0.01 **	0.01 ^ns^	0.23 ^ns^
Error	22	20,479.44	503.32	4.83	256.57	0.00002	0.002	0.0007	0.10
C.V(%)		8.01	4.31	9.45	12.70	3.34	5.50	4.50	1.93

ns (not significant); ** (significant at *p* < 0.01); df (degrees of freedom); EO (essential oil); EOY (essential oil yield); FO (fixed oil); FOY (fixed oil yield); BY (biological yield); GY (grain yield).

**Table 4:** Some characteristics of poultry and animal manure and vermicompost used in the experiment.

Characteristics of Fertilizer	C (%)	N (%)	P (%)	K (%)
Poultry manure	4.5	1.75	2.8	2.15
Animal manure	2.9	2.27	0.94	1.25
Vermicompost	3.8	1.46	2.4	1.95

## Data Availability

Data are available from the corresponding author upon reasonable request.
